# Ongoing Dengue Epidemic — Angola, June 2013

**Published:** 2013-06-21

**Authors:** Ministry of Health of Angola, Angola Country Office, World Health Organization, Div of Global HIV/AIDS, Div of Global Health Protection (proposed), Center for Global Health, Div of Global Migration and Quarantine, Div of Vector-Borne Diseases, National Center for Emerging and Zoonotic Infectious Diseases, CDC

On April 1, 2013, the Public Health Directorate of Angola announced that six cases of dengue had been reported to the Ministry of Health of Angola (MHA). As of May 31, a total of 517 suspected dengue cases had been reported and tested for dengue with a rapid diagnostic test (RDT). A total of 313 (60.5%) specimens tested positive for dengue, including one from a patient who died. All suspected cases were reported from Luanda Province, except for two from Malanje Province. Confirmatory diagnostic testing of 49 specimens (43 RDT-positive and six RDT-negative) at the CDC Dengue Branch confirmed dengue virus (DENV) infection in 100% of the RDT-positive specimens and 50% of the RDT-negative specimens. Only DENV-1 was detected by molecular diagnostic testing. Phylogenetic analysis indicated this virus has been circulating in the region since at least 1968, strongly suggesting that dengue is endemic in Angola. Health-care professionals throughout Angola should be aware of the ongoing epidemic, the recommended practices for clinical management of dengue patients, and the need to report cases to MHA. Persons in Angola should seek medical care for acute febrile illness to reduce the risk for developing complications. Laboratory-confirmed dengue also has been reported from seven countries on four continents among persons who had recently traveled to Luanda, including 79 persons from Portugal. Angola is the third of four African countries to report a dengue outbreak in 2013. Persons returning from Africa with acute febrile illness should seek medical care, including testing for DENV infection, and suspected cases should be reported to public health authorities.

## Background

Luanda, the capital city of Angola, has a population estimated at 5–20 million. No census has been conducted in Angola for several decades, primarily because of civil war during 1975–2002. A large proportion of the residents of Luanda live in densely populated urban slums and tenement housing. Access to health care is limited. Luanda is visited by many international business travelers, primarily because of commerce in oil.

Weak centralized surveillance for illnesses of public health importance has made it difficult for MHA to focus resources on populations in need. Although malaria is the greatest cause of morbidity and mortality in Angola (1), incidence is comparatively low in Luanda (2); however, an increase in malaria cases was detected in Luanda in 2012. Dengue was reported in travelers recently returned from Angola in 1986 and during 1999–2002 (3). Surveys conducted by the National Malaria Control Program during 2010–2012 showed that *Aedes aegypti* is the only DENV vector in Angola, and is present in all 18 provinces except Moxico.

## Epidemiologic and Laboratory Investigation

**FIGURE 1:**
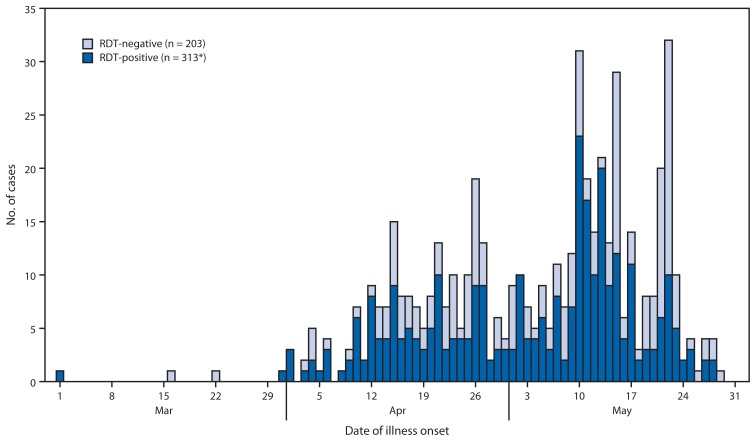
Number of reported dengue cases, by rapid diagnostic test (RDT) status and date of illness onset — Angola, March 1–May 31, 2013 * Two RDT-positive cases had no date of illness onset or specimen collection available.

Because routine surveillance for acute febrile illnesses, including dengue, is not well established in Angola, the number of reported fatal and nonfatal cases likely underestimates the actual number. Anecdotal reports from clinicians and residents of Luanda suggest that the number of nonmalaria acute febrile illnesses increased beginning in late January 2013, at which time dengue was included in the differential diagnosis. Testing of these cases with an RDT (Dengue Duo, Standard Diagnostics) that detects DENV nonstructural protein 1 (NS1) and anti-DENV immunoglobulin M (IgM), at the National Public Health Institute identified the first reported RDT-positive case with illness onset on March 1 (Figure 1).

The numbers of RDT-positive and RDT-negative cases began to increase noticeably in early April. A total of 517 suspected dengue cases had been reported to the National Public Health Institute with illness onset dates through May 31, of which 313 (60.5%) had specimens with RDT-positive results. RDT-positive patients were aged 0.8–77 years (median: 25 years), and 184 (59.9%) were male. Two suspected dengue cases were reported from outside Luanda Province, both from Malanje Province, including one with a specimen that was RDT-positive. Although detailed clinical information is unavailable for reported cases, one RDT-positive case has been reported with clinically significant hemorrhagic manifestations (e.g., hematemesis). In addition, one fatal RDT-positive case has been reported; however, anecdotal reports from clinicians and the public suggest that additional fatal cases occurred but were not reported to MHA.

**FIGURE 2:**
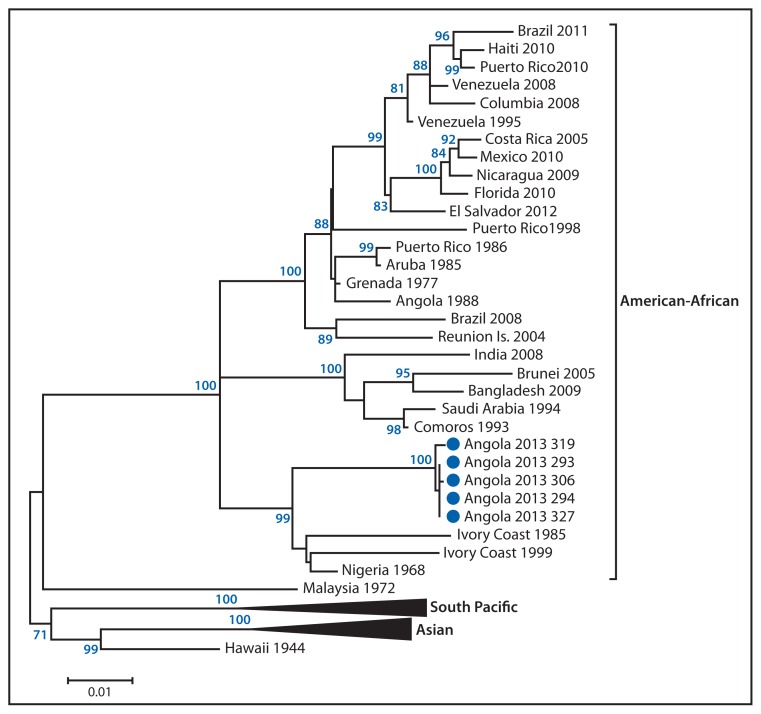
Phylogenetic tree* depicting the dengue virus-type 1 circulating in Angola in 2013 * Blue dots indicate viruses circulating in Angola in 2013 for which the envelope gene was sequenced; blue numbers reflect the bootstrap percentage likelihood that the indicated node exists.

Serum specimens from 49 suspected dengue cases with RDT results from the National Public Health Institute were sent to the CDC Dengue Branch for confirmatory diagnostic testing. Of these 49 specimens, 43 were RDT-positive (41 NS1-positive, 14 IgM-positive, and 12 positive for both NS1 and IgM) and six were RDT-negative. All specimens were tested by real-time reverse transcriptase–polymerase chain reaction (rRT-PCR) (DENV 1–4 Real-Time RT-PCR Assay, CDC) and immunoassay for anti-DENV IgM (DENV Detect IgM Capture ELISA, InBios International). Specimens testing NS1-positive only by RDT and not confirmed by rRT-PCR were submitted to an NS1 test (Panbio Dengue Early ELISA, Alere). Current or recent DENV infection was confirmed in all of the RDT-positive specimens and in three of the RDT-negative specimens. Only DENV-1 was detected by rRT-PCR. Direct nucleic acid sequencing from five serum specimens and subsequent phylogenetic analysis showed that the DENV-1 currently circulating in Luanda belongs to the American-African lineage (Figure 2). The closest identified ancestor of the virus was isolated from a specimen collected in Nigeria in 1968.

## Entomologic Investigation

Household surveys of container-breeding mosquitoes were conducted throughout Luanda. A total of 862 households were surveyed, of which 385 (44.7%) had at least one container with mosquito larvae present. Of 3,103 containers examined, 724 (23.3%) were colonized by mosquitoes. Most (63.1%) colonized containers were found indoors, and most were uncovered water-storage containers. The predominant mosquito species identified was *Aedes aegypti*.

## Public Health Response

Public health messages to alert the population of Luanda to the epidemic have been issued since April. Because public awareness of dengue in Angola is low, messaging has focused on the signs and symptoms of dengue, including how to identify warning signs of severe disease. The public also has been made aware of the need to clean up refuse and empty or cover water containers that can serve as mosquito breeding sites. Proposed biologic and chemical vector-control measures to be conducted by the National Malaria Control Program include fumigation to kill adult mosquitoes using organophosphates (fenitrothion or malathion), indoor residual spraying of households, and treating larval habitats with *Bacillus thuringiensis israelensis*.

MHA worked with teams from the World Health Organization and CDC, each composed of one epidemiologist and one entomologist, to guide the public health response to the epidemic. Activities included conducting a rapid assessment of mosquito populations in Luanda, improving clinical awareness of dengue and patient management by conducting training for health-care professionals, and encouraging clinicians to use RDTs to diagnose suspected cases and send the results to the MHA. As a result, increases in case reporting were observed starting in mid-May.

### Editorial Note

Recent data suggest that approximately 390 million DENV infections occurred worldwide in 2010, of which 96 million resulted in symptomatic illness (4). Most persons with symptomatic DENV infection will experience an acute febrile illness characterized by fever; headache; joint, muscle, and eye pain; and minor hemorrhagic manifestations (e.g., petechiae and epistaxis) that will resolve within 1 week with bed rest, oral rehydration, and avoidance of aspirin and nonsteroidal anti-inflammatory medications (5). Approximately 5% of persons with symptomatic infections can experience severe manifestations around the time of defervescence because of an increase in vascular permeability leading to plasma leakage and the potential for clinically significant pleural effusions and ascites, hypovolemic shock, severe hemorrhage (e.g., hematemesis and melena), and death. The primary factors that affect the case-fatality rate for severe dengue, which can range from <0.1% to 5%, are the timing and quality of clinical care that patients receive. Life-saving care depends on close monitoring of hemodynamic status and judicious use of intravenous fluids, especially in the 24–48 hours after fever has resolved (5).

Reporting of suspected and RDT-positive dengue cases should continue to be strengthened in Angola. However, because of deficiencies in the national reporting system, direct communication between hospitals and MHA might be the most effective means to monitor dengue activity. Of particular concern from a surveillance viewpoint is the low number of reported fatal cases, given the size of the population of Luanda and lack of experience with clinical management of severe dengue. The apparent lack of fatal case reporting might be explained by deaths that occurred outside the hospital, lack of accurate case diagnosis, lack of postmortem tissue to diagnose suspected dengue-related deaths, and lack of familiarity with how to report cases to MHA.

At least 91 laboratory-confirmed dengue cases have been reported recently in seven countries (Canada, France, Germany, Israel, Portugal, South Africa, and the United States) among persons who had recently traveled to Luanda (6,7). On May 24, CDC posted a travel notice on the Travelers’ Health website,* informing travelers and U.S. citizens living in Angola of the current dengue epidemic and reminding them to employ mosquito avoidance strategies and seek medical care for dengue-like illness. Four countries (Seychelles, Kenya, Angola, and Tanzania) thus far have reported dengue outbreaks in 2013. If travelers to Africa develop signs or symptoms of dengue during or ≤14 days after their visit, they should seek medical treatment and inform their doctor of their recent travel. Clinicians in the United States are reminded that dengue is a nationally reportable condition, and cases should be reported to local public health authorities. Clinicians can obtain dengue diagnostic testing from several national testing laboratories or state public health laboratories. Residents of and travelers to areas with endemic dengue can reduce their risk for DENV infection by using mosquito repellent, wearing long-sleeved shirts and pants, and sleeping in locations with air conditioning or screens on doors and windows. Up-to-date, destination-specific dengue activity reports can be found on CDC’s DengueMap.† Additional information on dengue and dengue prevention activities can be found at the CDC dengue site.§

What is already known on this topic?

Dengue is believed to be endemic in much of Africa, where an estimated 64 million dengue virus (DENV) infections occurred in 2010. Dengue has been documented previously in travelers returning from Angola, but information on the epidemiology of dengue in Angola has not been available.

What is added by this report?

This report documents an ongoing dengue epidemic in Angola that at present appears to be primarily affecting Luanda. Only DENV-1 has thus far been detected, and phylogenetic analysis indicated that the most closely related virus was isolated in Nigeria in 1968, demonstrating that this virus has been circulating in the region for at least 45 years and strongly suggesting that dengue is endemic in Angola.

What are the implications for public health practice?

Physicians and public health professionals should be aware that dengue is endemic in Angola and throughout much of Africa and that timely initiation of care of dengue patients can be life-saving. Clinicians in Africa and those examining patients with acute febrile illness and recent travel to Africa should suspect dengue and report cases to public health authorities.

The molecular phylogeny of the DENV-1 currently circulating in Luanda indicates that the virus likely has been circulating in the region since at least 1968. This finding, in combination with reports of dengue in travelers to Angola since the 1980s, strongly suggests that dengue is endemic in Angola, as it is in much of the rest of Africa (3,4). In support of these observations, a recent study predicted that approximately 16% of all DENV infections worldwide occur in sub-Saharan Africa (4). This suggests that dengue in Africa is on par with that of the Americas, where the widespread nature of the illness is recognized. Public health and health-care professionals should be aware that dengue is endemic in Africa and should test suspected cases with available diagnostic tests, including RDTs, and ensure that test results are confirmed at reference laboratories experienced in dengue diagnostic testing. Diagnostic and confirmatory testing should be performed to confirm cases and initiate appropriate clinical treatment, enable early detection of outbreaks or epidemics, and identify the DENV-types circulating in the region.

## Footnotes

* Available at http://wwwnc.cdc.gov/travel/notices/watch/dengue-angola.

† Available at http://www.healthmap.org/dengue.

§ Available at http://www.cdc.gov/dengue.
